# Implementation of a standardized Video‐based Asynchronous Neurological Examination (VANE) in a multi‐center study of AD/ADRD: Findings from The Diabetes Prevention Program Outcomes Study (DPPOS) AD/ADRD Project

**DOI:** 10.1002/alz.086250

**Published:** 2025-01-09

**Authors:** James M. Noble, Neelesh Nadkarni, Dianilka Martinez, Marinella Temprosa, Anna Bowers, Lindsay Doherty, Gerardo J Febres, Danurys Sanchez, Terry E. Goldberg, Hanna Sherif, Jose A. Luchsinger, Diabetes Prevention Program Research Group

**Affiliations:** ^1^ Columbia University Irving Medical Center, New York, NY USA; ^2^ University of Pittsburgh, Pittsburgh, PA USA; ^3^ Columbia University Medical Center, New York, NY USA; ^4^ George Washington University, Washington, DC USA

## Abstract

**Background:**

The Diabetes Prevention Program Outcomes Study (DPPOS) is an established cohort of aging persons (mean age 72 years) with prediabetes and diabetes with a mean of 23 (range 21‐25) years of follow‐up. DPPOS added neuropsychological testing using the National Alzheimer’s Coordinating Center (NACC) Uniform Data Set (UDSv3) forms. Using the NACC UDS required implementing a standardized neurological examination across 25 US clinical sites, administered by project coordinators (PC).

**Methods:**

A video‐based asynchronous neurological examination (VANE) was developed by clinicians with experience in the NACC‐UDS to harmonize with UDSv3 to anticipate common neurological diagnoses aside from dementia including diabetic cranial neuropathy, stroke and Parkinsonism. VANE was designed to be contactless, reproducible, to be administered by non‐clinicians, such as PCs. Using standard iPad software connected to a secure repository, the video exam includes extraocular and facial movements, visual field, speech (“Cookie‐theft”), gross motor strength, pronator drift, praxis and Parkinsonism (i.e. the Unified Parkinson’s Disease Rating Scale modeled examination as in UDSv3, including assessment of tremors, bradykinesia, finger and foot tapping, hand movements, postural stability and gait). Rigidity and pull‐testing were not included. A 10‐minute training video was developed for study staff, which demonstrates the examination step‐by‐step with scripts and video instructions in English and Spanish. Site‐specific performance review, feedback, and staff certification preceded central reading of video recordings by three physicians.

**Results:**

During calendar year 2023, 608 neurological examinations occurred at 25 sites, each lasting 10 to 15 minutes. Each site on average completed 24 examinations (SD = 13.0, range 3‐48). Of the initial 442 examinations reviewed, 406 (91%) were completed using the script provided, 421 (95%) were error‐free, and 3 (0.6%) were non‐interpretable. Abnormal findings were noted in 153 participants (2% Parkinsonism, 1% cerebrovascular disease and 31% other neurological abnormalities). Suboptimal exams occurred at 10 of 25 sites (range 1‐4 per site) early in implementation.

**Conclusions:**

Engaging research staff across 25 sites, and three physician‐reviewers, this study is the first to implement VANE as an efficient neurological examination model. VANE presents a novel paradigm for large multisite epidemiological studies considering incorporating neurological examinations conducted by non‐clinicians.

